# Combined use of circulating tumor cells and salivary mRNA to detect non–small-cell lung cancer

**DOI:** 10.1097/MD.0000000000019097

**Published:** 2020-02-21

**Authors:** Xianwen Gu, Junfeng He, Guanglei Ji

**Affiliations:** a The First Department of Thoracic Surgery; b The Second Department of Abdominal Surgery, Linyi Cancer Hospital, Shandong, China.

**Keywords:** circulating tumor cell, lung cancer, mRNA, non–small-cell lung cancer, saliva

## Abstract

Liquid biopsy is an emerging technique for noninvasive detection of various cancers. Majority of liquid biopsy tests still, however, use solitary type of biomarkers with unsatisfactory sensitivity and specificity. To this end, a combined approach of circulating tumor cells (CTCs) and salivary mRNA biomarkers was evaluated for discriminating non–small-cell lung cancer (NSCLC) from healthy controls.

Our study included a discovery phase to find multiple biomarkers, and an independent validation phase to confirm the applicability of the selected biomarkers. In the discovery phase, CTC level in blood and 5 mRNA biomarkers in saliva (i.e., CCNI, Epidermal growth factor receptor [EGFR], FGF19, FRS2, and GREB1) were measured for 140 NSCLC patients and 140 healthy controls, followed by developing a predictive model. Next, this panel of biomarkers was applied to another patient cohort consisted of 60 patients with NSCLC and 60 healthy controls in the validation phase.

We found that our novel biomarker panel could differentiate patients with NSCLC from healthy controls with high sensitivity (92.1%) and high specificity (92.9%) in the discovery phase. In the validation phase, we achieved sensitivity of 88.3% and specificity of 90.0%.

To our best knowledge, it is the first time that a combined use of CTC and salivary mRNA biomarkers were applied for noninvasive detection of NSCLC.

## 1 Introduction

Lung cancer is estimated be the most commonly diagnosed cancer (2.09 million cases) and the leading cause of cancer mortality (1.76 million deaths) in the world in 2018.^[1]^ Lung cancer includes non–small-cell lung cancer (NSCLC) and small-cell lung cancer, with the NSCLC accounting for >80% of lung cancer cases. The overall 5-year survival rate of lung cancer is 18% for all stages, but the 5-year survival rate for distant lung cancer is much lower (5%), according to the American Cancer Society.^[2]^ Such high death rate is largely attributed to the difficulty of early cancer detection.

Circulating tumor cell (CTC) is a promising blood-based liquid biopsy method for detecting multiple cancers.^[3]^ CTCs are cells that have shed from a tumor into the blood,^[4]^ and can be detected via cytokeratin staining. The first Food and Drug Administration–cleared commercial CTC test is CellSearch,^[5]^ in which immunomagnetic nanoparticles are directed against the epithelial cell adhesion molecule to isolate and concentrate epithelial tumor cells for CTC detection. The isolation of CTCs can, however, be technically challenging, since CTC can be extremely rare in blood samples (e.g., 1 CTC out of millions to billions of blood cells in the background).^[6–8]^

Salivary mRNA is recently proposed as another, highly promising liquid biopsy method. Analysis of salivary mRNA biomarkers have been conducted for many cancers, such as lung cancer,^[9]^ gastric cancer,^[10]^ breast cancer,^[11]^ pancreatic cancer,^[12]^ ovarian cancer,^[13]^ and oral squamous cell carcinoma.^[14–17]^ For lung cancer in particular, CCNI, FGF19, GREB1, EGFR, and FRS2 were successfully applied to differentiate lung cancer patients from normal control subjects, with 93.75% sensitivity and 82.81% specificity in a prevalidation sample phase of 32 patients with cancer and 64 healthy controls.^[9]^ These salivary RNA biomarkers were, however, mostly validated for American populations.^[9–18]^ It remains unclear whether or not these biomarkers can also be applied for other populations.

So far, most of the liquid biopsy studies on cancer detection were conducted by using either blood biomarkers such as CTC or salivary biomarkers such as mRNA biomarkers. This study aimed at exploring the potential of combining both blood biomarkers and salivary biomarkers to detect lung cancer. To this end, we measured the CTC levels in blood and mRNA expression levels of CCNI, EGFR, FGF19, and FRS2 in saliva of 140 NSCLC patients and 140 healthy controls, respectively. We then developed a predictive model for sample classification, which achieved both high sensitivity (92.1%) and high specificity (92.9%). To further test the applicability of our biomarker panel, we recruited an independent patient cohort of 60 patients with NSCLC and 60 healthy controls, blinded the samples, and applied the biomarker panel to make predictions of NSCLC occurrence. We successfully differentiate patients with NSCLC from healthy controls with sensitivity of 88.3% and specificity of 90.0%. Overall, this study confirmed that the combined use of blood biomarkers and salivary biomarkers could improve the detection accuracy and presents a promising clinical approach as a noninvasive detection approach for lung cancer detection.

## 2 Materials and methods

### 2.1 Participants of this study

This study was approved by the Linyi Cancer Hospital. Two phases of study were designed and conducted: a biomarker discovery phase and an independent validation phase (Table 1). In the discovery phase of biomarkers, our examined population consisted of 140 patients with NSCLC from Linyi Cancer Hospital. These patients were recruited between April 2, 2015 and January 8, 2016. As healthy controls, 140 non-NSCLC people were included as the blood and saliva donors in the same period of time. In the validation phase, we followed a similar procedure to recruit 60 patients with NSCLC and 60 healthy controls from February 16, 2016 to January 15, 2017. For all research participants, we obtained written-informed consent forms.

**Table 1:**
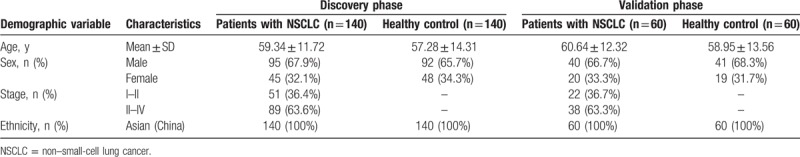
Demographic information of all subjects used in this study.

### 2.2 CTC analysis in blood samples

CTC enumeration was conducted as below and the same procedures were applied for participants in both the biomarker discovery phase and the validation phase. Briefly, a total of 5 mL whole blood samples were collected in 10-mL tubes with acid citrate dextrose–anticoagulant (Becton Dickinson, NJ) to collect CTCs. Sera were separated, aliquoted, and stored at −25°C until using imFISH assays for CTC enumeration. These procedures were finished within 30 minutes after collection. We then followed a previously described method (30) to conduct imFISH assay. In short, cells were separated from whole blood via centrifugation from 4 mL collected blood, followed by red blood cell hypotonic hemolysis. phosphate buffered saline was then used to resuspend the residual cell particles, followed by incubating cell particles with anti-CD45 monoclonal antibody-coated magnetic beads (Life Technologies, Carlsbad, CA) for 30 minutes and wiping out the magnetic beads loaded with the majority of leukocytes by a magnetic stand (Promega, Madison, WI) from the cell suspension. Immunofluorescence analysis was next conducted using supernatants. CTCs of lung cancer were identified by negative enrichment and immune fluorescence in situ hybridization (NEimFISH). In brief, anti-CD45 monoclonal antibody (red) and FISH with chromosome 8 (orange) centromere probe (CEP8; Abbott Molecular Diagnostics, Des Plaines, IL) were combined in NEimFISH. CEP8 probe and specimen were hybridized in hybridizer (DAKO) at 37°C for 20 minutes, followed by washing in 50% formamide at 43°C for 15 minutes, and immersing into 2 × saline sodium citrate and gradient alcohol. Then, 0.2% bovine serum albumin (BSA) was used to wash the specimens twice, followed by incubating the specimens with the CD45 mixture/2% BSA conjugated to Alexa Fluor 594 Invitrogen (Carlsbad, CA, USA) for 1 hour. 0.2% BSA was next used again to wash the specimens. At last, 4’,6-diamidino-2-phenylindole (DAPI) that contained Vectashield mounting medium was used to cover the specimens. The samples were observed along the “S” track with a microscope (Nikon). Positive CTCs were stained as CEP8+/DAPI+/CD45−.

### 2.3 mRNA level measurement in saliva samples

The levels of mRNA in saliva samples were quantified as below and the same procedures were applied for participants in both the biomarker discovery phase and the validation phase. In brief, the Oragene Self-Collection Kit OG-500 (DNA Genotek Inc., Ottawa, Ontario, Canada) was used to collect saliva from participants by following the manufacturer's protocol. The participants were instructed to have no eating/drinking/smoking/oral hygiene procedures for at least 1 hour before sample collection. Totally 2 mL whole saliva was collected and placed on ice. To avoid degradation of salivary RNA, we centrifuged saliva samples at 15,000 g at 4°C for 9 minutes, removed the supernatant, treated it with RNase inhibitor (Superase-In, Ambion Inc., Austin, TX) and stored it at −80°C. Then, a previously published protocol was used to extract salivary RNA.^[9]^ In short, 330 μL saliva supernatant was used to extract RNA with RNeasy Protect Saliva Mini Kit (Qiagen, Germany), according to manufacturer's protocol. The salivary RNA was then treated with TURBO DNase treatment and used as template for cDNA synthesis. Quantitative real-time polymerase chain reaction was next performed by using Roche LightCycler 480 (Roche, Switzerland). The volume of reactions was set as 20 μL, which contained primers (Table 2), cDNA, the probe, and the reaction mix. We strictly followed the manufacturer's protocol and used Glyceraldehyde 3-phosphate dehydrogenase (GAPDH) as our internal control. The Ct value was then used to calculate relative mRNA level, as described previously.^[19]^ In sum, ΔCt value was generated by normalizing the mRNA level to Ct of GAPDH. The level of mRNA was quantified as 2^(− ΔCt)^ multiplied by 1000.

**Table 2:**
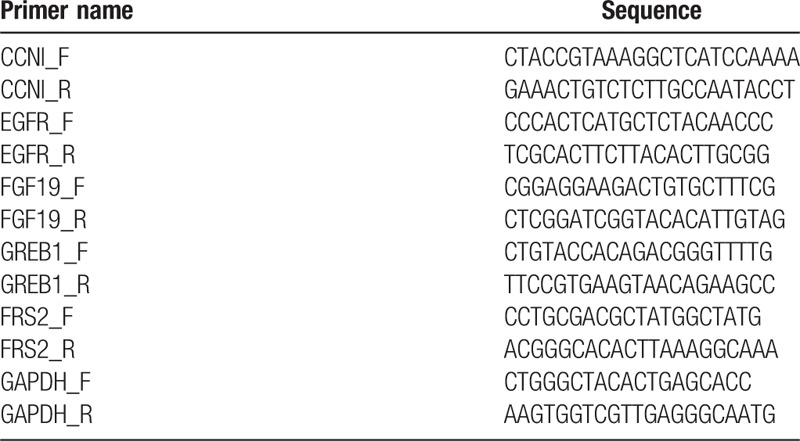
Primers used in this study.

### 2.4 Statistical analysis and machine learning–based classification

Comparison of the CTC enumeration and mRNA level between the patient group and the control group was conducted by independent sample *t* test. We chose *P* value <.05 as statistically different. We further applied the receiver operating characteristic (ROC) curve for each biomarker and calculated the corresponding area under the curve (AUC). This allowed us to evaluate the discriminatory power of each biomarker. All of the statistical analysis was performed using MedCalc (MedCalc, Belgium). A panel of selected biomarker that had AUC value >0.70 was identified for classification analysis. We chose logistic regression as our classifier for data collected in the biomarker discovery phase. The same algorithm has been found to be effective in other liquid biopsy studies.^[20]^ We used R glmnet package to perform the logistic regression, and set lambda parameter to zero. To avoid overfitting, we also conducted 10-fold cross-validation in the datasets. The trained classifier was next applied to the data collected in the validation phase. In brief, we predicted the occurrence of NSCLC by using the classifier and compared our predictions with the diagnosis. Sensitivity and specificity were calculated correspondingly to evaluate the prediction performance.

## 3 Results

### 3.1 Overview of study design

**Figure 1:**
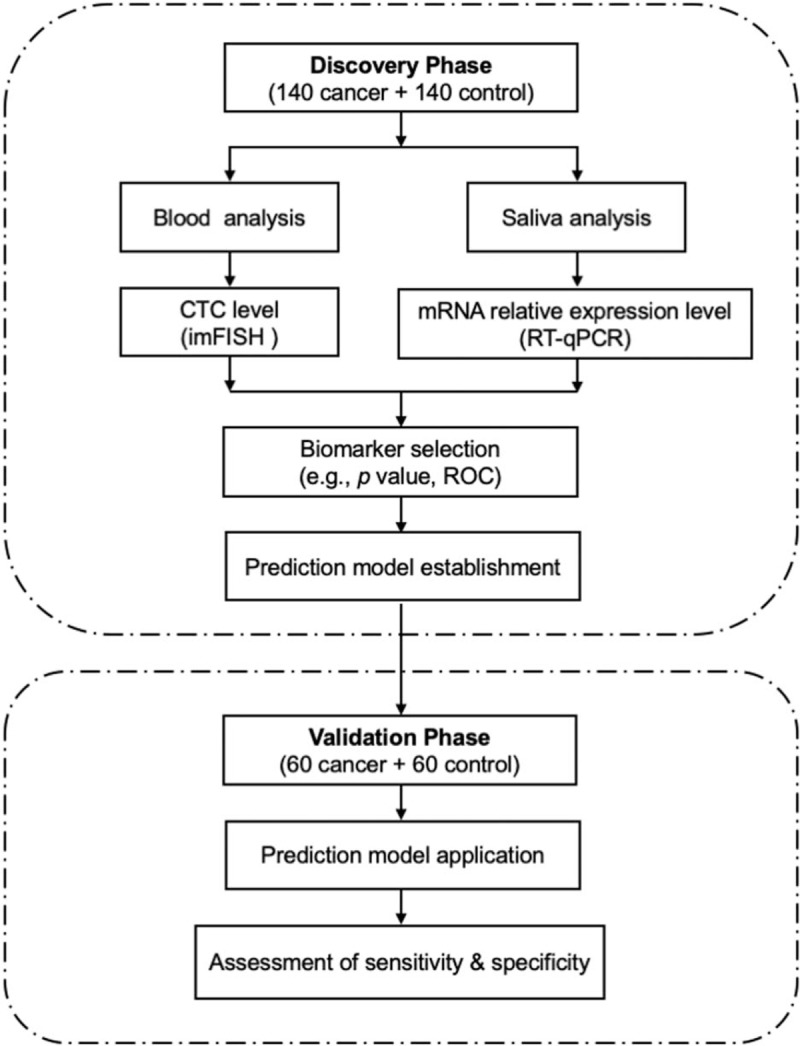
Schematic diagram of the study design to develop a biomarker panel for non–small-cell lung cancer (NSCLC) detection. CTC = circulating tumor cell, ROC = receiver operating characteristic, RT-qPCR = Quantitative real-time polymerase chain reaction.

This study was designed to include 2 phases: a biomarker discovery phase and an independent validation phase (Fig. 1). The biomarker discovery phase aims to measure and evaluate candidate biomarkers from blood and saliva for developing a predictive approach for classification of patients with NSCLC. We recruited a total of 140 patients with NSCLC and 140 healthy controls in this phase and for each participant, we measured the CTC level in blood samples and expression levels of candidate genes in saliva samples. We next developed a machine learning–based model to predict NSCLC occurrence. After discovering the biomarker panel, we would like to further evaluate its applicability in clinical detection of NSCLC. Therefore, we designed the independent validation phase and recruited a separate patient cohort of 60 patients with NSCLC and 60 healthy controls. In the validation phase, we blinded the samples and measured the biomarker levels in corresponding samples, and made predictions on whether or not a sample was from a patient with NSCLC. We compared our predictions with pathological classification and calculated sensitivity and specificity to evaluate the clinical performance of our method.

### 3.2 Measurement and comparative analysis of biomarker levels in the discovery phase

**Figure 2:**
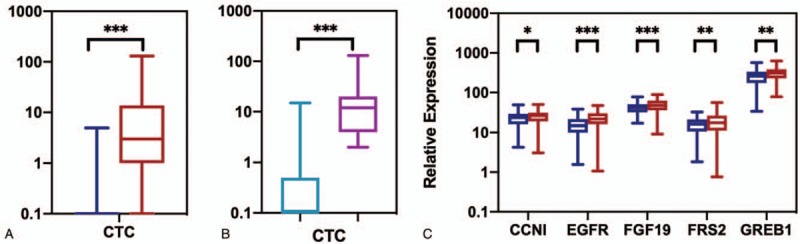
Analysis of biomarkers in training datasets. A, CTC level of blood samples in patients with non–small-cell lung cancer (NSCLC) (red) and healthy controls (blue). B, CTC level of blood samples in patients with early-stage NSCLC (light blue) and patients with late-stage NSCLC (purple). C, The relative expression levels of mRNAs of saliva samples in NSCLC patients (red) and healthy controls (blue). ^∗^*P* < .05; ^∗∗^*P* < .01; ^∗∗∗^*P* < .001. CTC = circulating tumor cell.

We measured 2 types of biomarkers for each participant of the patient cohort in the discovery phase (consisted of 140 patients with NSCLC and 140 healthy controls): the CTC levels in blood and the expression levels of 5 mRNA biomarkers in saliva (i.e., CCNI, EGFR, FGF19, FRS2, and GREB1). We then compared the biomarker level between the patients with NSCLC and healthy controls. For CTC biomarker in blood (Fig. 2A), we found that the CTC level was significantly elevated for patients with NSCLC (i.e., mean CTC = 0.08 for healthy controls and mean CTC = 9.79 for patients with NSCLC, *P* < .001). We also found that the difference of CTC level between patients with early-stage (stage I–II) NSCLC and patients with late-stage (stage III–IV) NSCLC was significant (i.e., mean CTC = 2.07 for patients with early-stage NSCLC and mean CTC = 13.23 for patients with late-stage NSCLC, *P* < .001) (Fig. 2B). For mRNAs biomarkers in saliva (Fig. 2C), we found that all biomarkers demonstrated elevated expression levels in the patients with NSCLC (*P* < .05) compared to that of healthy controls. Among the 5 salivary mRNA biomarkers, EGFR showed highest elevation with a 1.45-fold increase of expression level in patients with NSCLC (*P* < .001).

### 3.3 Development of predictive models for discrimination of patients with NSCLC from healthy controls

**Figure 3:**
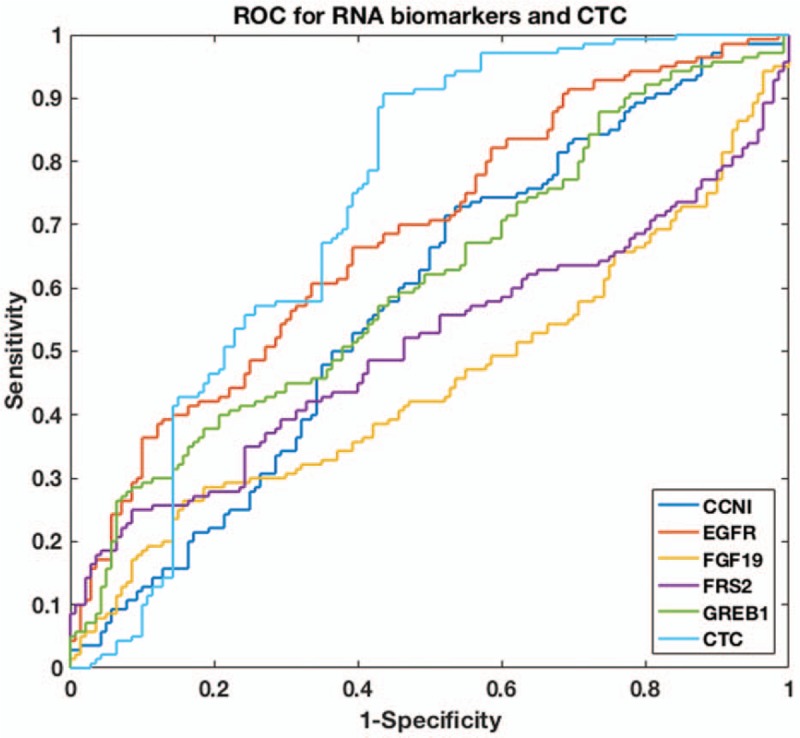
ROC curve of CTC and salivary mRNAs for non–small-cell lung cancer NSCLC classification. CTC = circulating tumor cell, ROC = receiver operating characteristic.

We first evaluated each biomarker's discriminatory power in separating patients with NSCLC from healthy controls by using ROC curve and calculating the AUC value (Fig. 3). This step is necessary as we found that certain biomarker only demonstrated moderate difference between patients with NSCLC and healthy controls. For example, the top one mRNA biomarker, EGFR, only showed <2-fold increase of expression level in patients with NSCLC. Although the levels of biomarkers were statistically different between patients with NSCLC and healthy controls, their discriminatory power needs to be rigorously evaluated. Based on previous studies on biomarker evaluation,^[21,22]^ we chose AUC value of 0.70 as our cut-off value, that is, if a biomarker has an AUC value >0.70, it is suggested to have a decent performance for discriminating patients with NSCLC from healthy controls. For the CTC biomarker in blood and the 5 mRNA biomarkers in saliva, CTC biomarker, and EGFR were the top 2 biomarkers with highest AUC values (0.73 and 0.70, respectively). The AUC values of rest biomarkers did not exceed 0.70, which indicated using these biomarkers alone generated low discrimination accuracy for patients with NSCLC.

**Figure 4:**
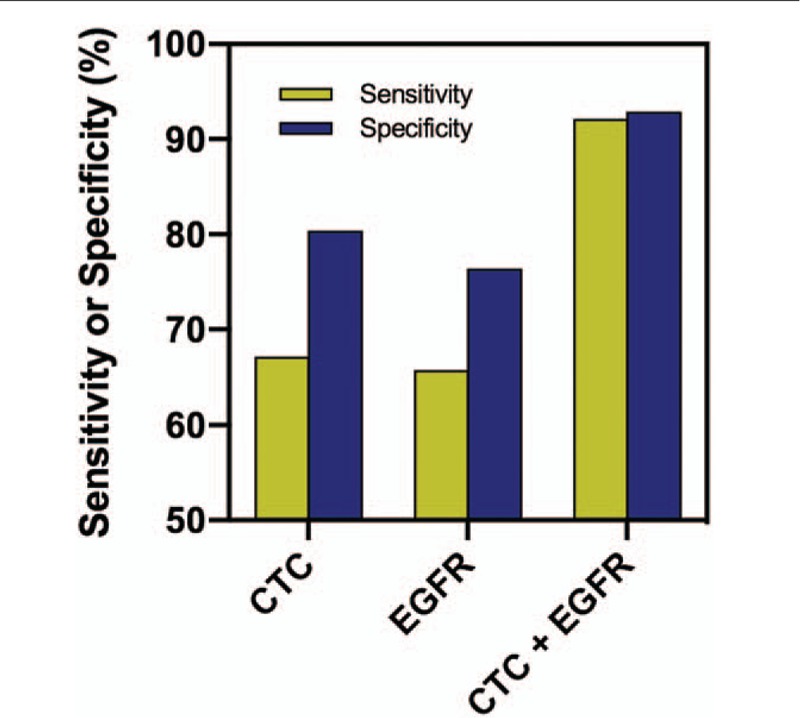
Performance of different biomarker panels in non–small-cell lung cancer (NSCLC) detection in the discovery phase. CTC = circulating tumor cell, EGFR = Epidermal growth factor receptor.

Because only 2 biomarkers, CTC and EGFR, have AUC values larger than the cut-off value, we then used these 2 biomarkers as our panel and built a machine learning algorithm for NSCLC classification. We chose logistic regression as our classifier and used the data collected in the biomarker discovery phase as our training database. It is worth mentioning that the same algorithm has been found to be effective in other liquid biopsy studies such as CancerSEEK.^[20]^ We used R package to perform the logistic regression and performed 10-fold cross-validation in the training database to avoid overfitting. As shown in Figure 4 and Table 3, our predictive model successfully separate patients with NSCLC from healthy controls, as the sensitivity reached 92.1% and specificity reached 92.9%. In comparison, using solitary biomarker of CTC or EGFR only led to sensitivity of 65.7% to 67.1% and specificity of 76.4% to 80.0%. This clearly suggested that combining different types of biomarkers could dramatically improve the accuracy in detection of NSCLC.

**Table 3:**
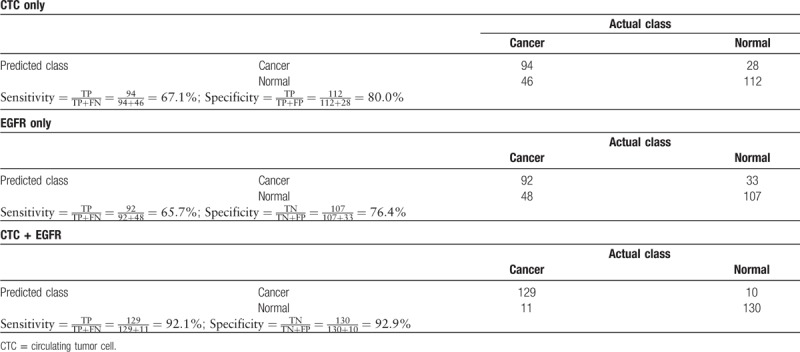
Confusion matrix of combined biomarker panel using circulating tumor cell and EGFR in the discovery phase.

### 3.4 Further evaluation of biomarker panel using independent validation datasets

**Figure 5:**
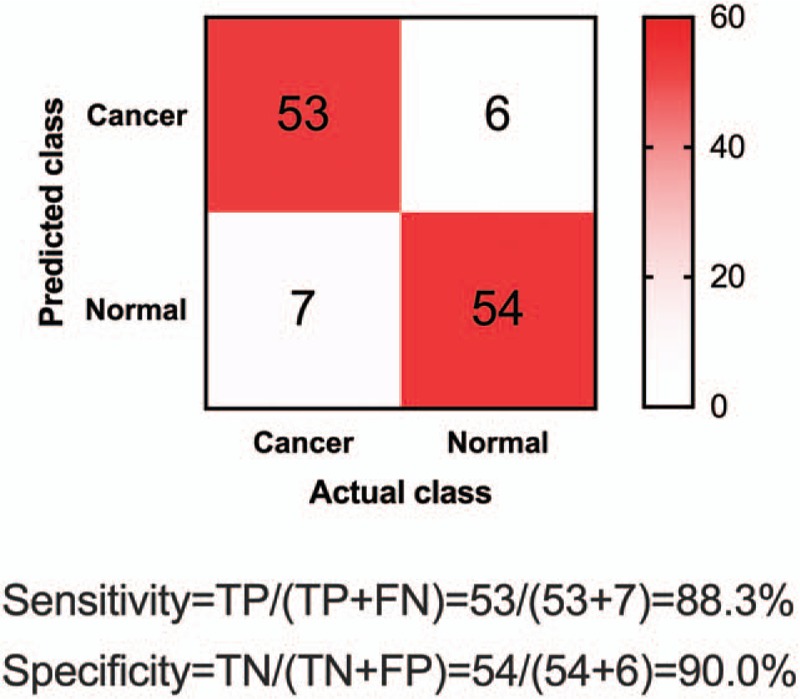
Confusion matrix of combined biomarker panel using circulating tumor cell (CTC) and EGFR in the independent validation phase.

We believe it is critical to evaluate our biomarker panel and test its applicability in independent validation datasets. Therefore, we designed the phase of validation study by recruiting another patient cohort of 60 patients with NSCLC and 60 healthy controls. The characteristics of the patient cohort in the validation phase were similar as that in the discovery phase (Table 1). We blinded the samples collected from the patient cohort, measured CTC level in blood and EGFR expression level in saliva, and then applied our model to predict whether or not a sample was from a patient with NSCLC. After prediction, we compared our results with pathological classification. We found that our predictions achieved high sensitivity of 88.3% and high specificity of 90.0% (Fig. 5). This strongly supported the conclusion that our combined biomarker panel of CTC and salivary mRNA could be a promising, noninvasive approach in clinical detection of NSCLC.

## 4 Discussion

This study proves the principle that combined use of CTC level and salivary mRNA level could achieve much higher sensitivity and specificity than solitary use of biomarker in detection of NSCLC. Possible reason for such improvement could be attributed to the complementary nature of these 2 biomarkers. For example, CTC level was found to be generally low in healthy controls and high in patients with NSCLC. It provided valuable information on selecting true positives (i.e., patients with NSCLC). The issue of false negatives was, however, severe when using CTC level alone, as we found the sensitivity of using CTC analysis alone was only 67.1%. The patients with NSCLC that were, however, wrongly labeled as negatives by CTC analysis could be correctly identified by EGFR analysis, and hence, the combined use of CTC biomarker and EGFR biomarker significantly improved the detection sensitivity. We also want to point out that the combined analysis of multiple biomarkers attracts increasingly more attention in the field of liquid biopsy. Recent development of CancerSEEK,^[20]^ for example, applied multiple ctDNA mutation biomarkers and serum protein biomarkers to successfully detect cancers such as liver and ovary cancer with high sensitivity (>95%). Although using CancerSEEK for lung cancer detection only achieved sensitivity of 60%, we found in this study that choosing a different set of biomarkers for combined analysis could overcome this issue and dramatically increased both sensitivity (88.3%) and specificity (90.0%).

We would like to highlight the use of machine learning for development of predictive models in this study. In recent years, machine learning has been seen to be widely integrated into discovery and improvement of various biomarkers, including ctDNA,^[23]^ ctRNA,^[24]^ proteomics,^[25,26]^ and metabolomics.^[27]^ Indeed, we also found that by using machine learning algorithm, we dramatically increased our detection accuracy. It is important to notice that sometimes machine learning could overfit a model, leading to very high accuracy using the training datasets but poor performance when being applied for real-world datasets. To avoid overfitting, we specifically designed the independent validation phase, collected new data from separate patient cohorts, blindly tested the samples, and evaluated our predictions. Our results of the validation phase study agreed well with that of the discovery phase, which indicated that there was no overfitting of our predictive model.

Finally, we wanted to point out a few limitations of this study. The research focus of this study was to discover and validate a biomarker panel for NSCLC detection. Therefore, investigation of the molecular mechanisms of the biomarkers was not fully explored here because this topic is beyond the scope of this work. We do want to mention that Zhang et al^[9]^ also found that EGFR was a suitable biomarker for NSCLC detection in Caucasian people. Also, since our work was primarily a pioneering study on combined use of biomarkers, we have not yet explored the entire landscape of all possible biomarkers for NSCLC detection. This certainly laid the possibility that certain biomarkers that were not covered in this study, for example, KRAS, NOTCH1, STRN, and TP53,^[28–31]^ could also contribute to the improvement of NSCLC detection. We are fully aware of this issue, and currently seeking collaborations with researchers in the United States and India for conducting a multicenter, large-scale clinical biomarker study to comprehensively evaluate as many NSCLC-relevant biomarkers as possible.

In sum, we demonstrated that combining CTC biomarker in blood and mRNA biomarker in saliva could improve the detection accuracy of NSCLC. Our method stands as a promising, noninvasive, liquid-biopsy approach for potential clinical application.

## Author contributions

**Conceptualization:** Xianwen Gu, Guanglei Ji.

**Data curation:** Xianwen Gu, Junfeng He.

**Formal analysis:** Xianwen Gu, Junfeng He.

**Investigation:** Xianwen Gu, Junfeng He.

**Methodology:** Xianwen Gu, Junfeng He.

**Project administration:** Guanglei Ji.

**Resources:** Guanglei Ji.

**Software:** Guanglei Ji.

**Supervision:** Guanglei Ji.

**Visualization:** Guanglei Ji.

**Writing – original draft:** Xianwen Gu, Junfeng He, Guanglei Ji.

**Writing – review and editing:** Xianwen Gu, Guanglei Ji.
